# The complete chloroplast genome sequence of *Swertia japonica* (Schult.) Makino (Gentianaceae)

**DOI:** 10.1080/23802359.2023.2275335

**Published:** 2023-11-01

**Authors:** Watanabe Yoichi

**Affiliations:** Graduate School of Horticulture, Chiba University, Matsudo, Japan

**Keywords:** Japan, medicinal plant, phylogenetic relationships

## Abstract

*Swertia japonica* (Schult.) Makino is a traditional medicinal plant in Japan for which the chloroplast genome has not been previously reported. The complete chloroplast genome of *S. japonica* was determined using a high-throughput sequencing technique. The total length of the *S. japonica* chloroplast genome was 153,208 bp, and comprised a large single-copy region of 83,319 bp, and a small single-copy region of 18,375 bp, separated by a pair of 25,757 bp inverted repeat regions. A phylogenetic analysis, based on the obtained chloroplast genome, indicated that *S. japonica* is closely related to *S. diluta*, *S. franchetiana*, *S. kouitchensis*, *S. mussotii*, and *S. punicea*. The presented chloroplast genome will be useful for further taxonomic, pharmacological and evolutionary studies of *Swertia*.

## Introduction

*Swertia japonica* (Schult.) Makino (Makino 1910) is a biennial plant that is found across Japan and Korea. This species is a part of traditional Japanese medicine, and is commonly used for gastrointestinal diseases including nausea, gastroparesis, and gastric atony (Kimura and Sumiyoshi 2011). Moreover, the plant has potential for antipyretic, anthelmintic, tonic, anti-periodic, cathartic, asthma, leucorrhoea, analeptic, anti-inflammatory, and relaxing the pregnant uterus (Rai et al. 2016). The genus includes other species that are used for medicinal purposes, but the taxonomy of the genus remains unresolved due to polyphyly (Favre et al. 2010). Therefore, insights into the genomic features and phylogenetic relationships among *Swertia* species will prove valuable to the taxonomic revision of this genus. In this study, we report the complete chloroplast genome of *S. japonica* based on Illumina paired-end sequencing data. In addition, phylogenetic relationships of the genus *Swertia* and related genera were reconstructed by utilizing published sequences of related species.

## Materials and methods

**Figure 1.**
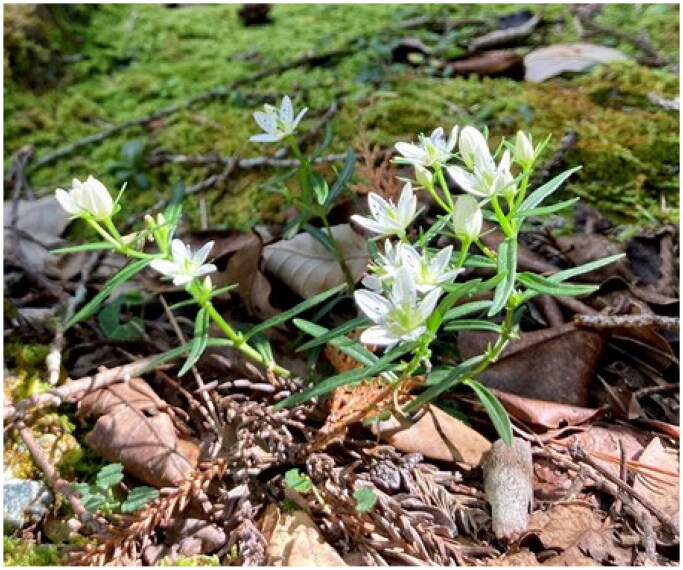
The analyzed sample of *Swertia japonica.* The photograph was taken by Watanabe Yoichi at Tsugeno, Aichi Prefecture, Japan, on 16 October 2022. *Swertia japonica* is a biennial plant 5–20 cm in height, with a corolla diameter of 2–3 cm and petal with nectaries.

Sample leaves were collected from Tsugeno in Aichi Prefecture, Japan (34.8639N, 137.5775E) on 16 October 2022 (GenBank BioSample, SAMD00578002). The sample was identified and collected by the author, and the voucher specimen was deposited in the Herbarium of the National Museum of Nature and Science, Japan (https://tbg.kahaku.go.jp/english/index.php, Atsushi Ebihara, ebihara@kahaku.go.jp) under the accession number TNS1347890 (Figure 1).

Total genomic DNA was extracted from dried leaves using the DNeasy Plant Mini kit (Qiagen, Hilden, Germany). The extracted DNA was used to prepare a sequencing library using the TruSeq DNA PCR-Free Kit (Illumina, San Diego, CA) and then sequenced using 150 base-length read chemistry in a paired-end flow cell on the NovaSeq 6000 platform (Illumina, San Diego, CA); the library preparation and sequencing were conducted by Macrogen (Seoul, South Korea). The 4.8 Gbases of raw data was trimmed by clipping adaptor sequences and removing reads with low quality using fastp v. 0.20.0 (Chen et al. 2018). Trimmed reads were deposited in the GenBank (accession number, DRR441012). Trimmed reads were assembled using GetOrganelle v.1.7.5 (Jin et al. 2020) with the following settings: -F embplant_pt; and default settings for other options. The assembled genome was annotated based on BLAST searches using GeSeq (Tillich et al. 2017) with default settings. To verify complete coverage of the chloroplast genome, the trimmed reads were mapped to the assembled genome using BWA (Li and Durbin 2009) with default settings. To facilitate the mapping to the circular genome, the sequence was linearized and duplicated 700 bp from one end to the opposite end. The genome map and cis/trans-splicing genes maps were drawn using CPGView (Liu et al. 2023). The annotated chloroplast genome was deposited in the GenBank (accession number, LC744566). To investigate the phylogenetic position of *S. japonica*, chloroplast genome sequences of related species were obtained from the GenBank database. A total of 23 *Swertia* species based on the research by Yang et al. (2022), and three *Gentiana* species, which served as outgroups of *Swertia*, were used. A total of 69 genes longer than 150 bp, which were shared among the species were extracted, and each orthologous gene was aligned using MAFFT v.7.505 (Katoh and Standley 2013) with default settings, and then these alignments were combined. A phylogenetic tree by the maximum likelihood method was constructed using RAxML v.8.2.0 (Stamatakis 2014) under the GTR + G + I substitution model with 1000 bootstrap replicates.

## Results

**Figure 2.**
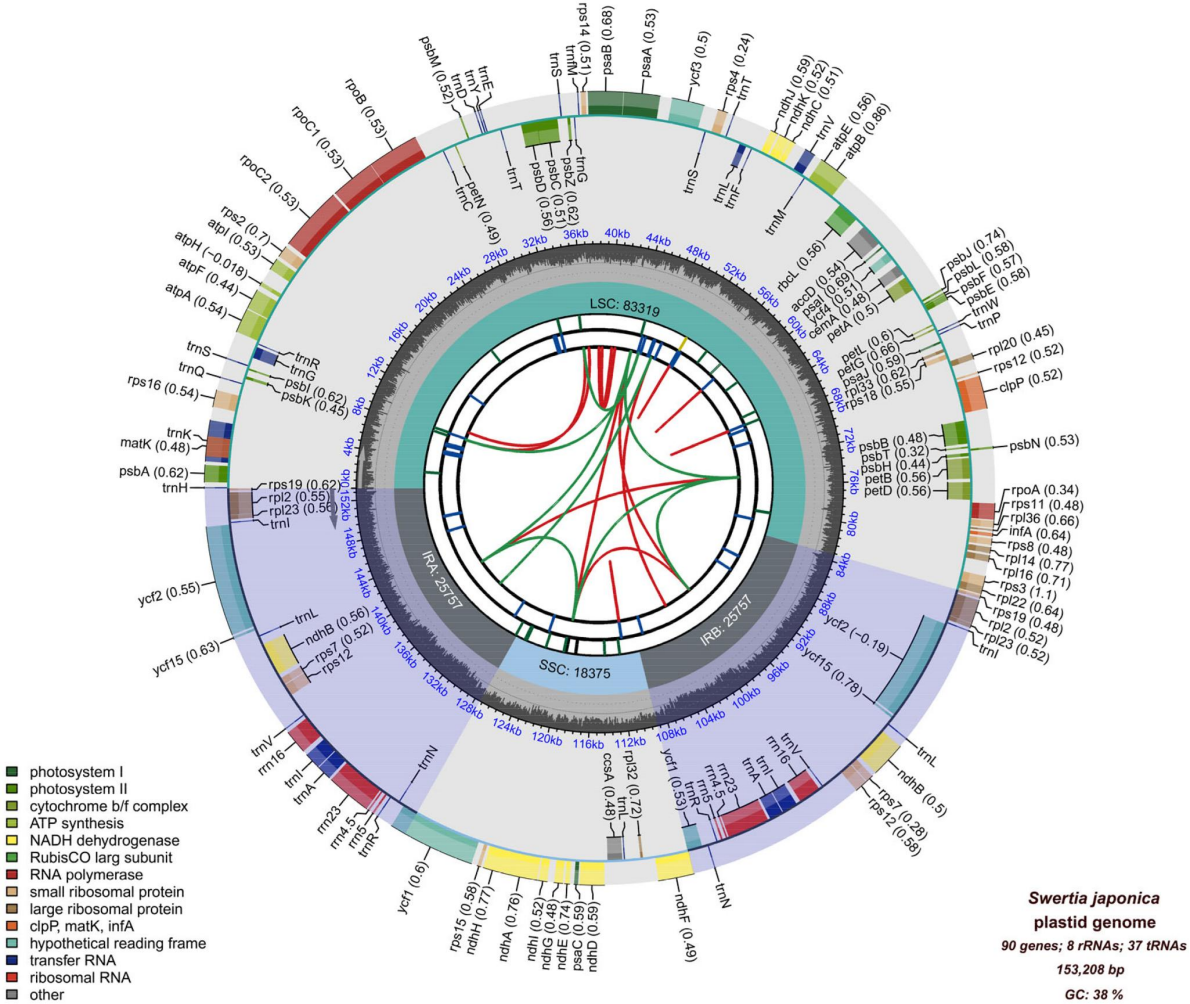
The complete chloroplast genome of *Swertia japonica*. The map contains six tracks. From the center outward, the first track displays the dispersed repeats. The dispersed repeats consist of direct and palindromic repeats, connected with red and green arcs. The second track shows long tandem repeats as short blue bars. The third track shows short tandem repeats or microsatellite sequences as short bars with different colors. The small single-copy (SSC), inverted repeat (IRA and IRB), and large single-copy (LSC) regions are shown on the fourth track. The GC content along the genome is plotted on the fifth track. The genes are shown on the sixth track. Optional codon usage bias is displayed in parentheses after the gene name. Genes belonging to different functional groups are color-coded. Genes on the inside and outside of the map are transcribed in clockwise and counterclockwise directions, respectively.

The total length of the complete chloroplast genome of *S. japonica* was 153,208 bp, with an average coverage of ×1578.9 and GC content of 38% (Figure 2, Supplemental Figure 1). A large single-copy (LSC: 83,319 bp), a small single-copy (SSC: 18,375 bp), and two inverted repeat (IR: 25,757 bp) regions made up the quadripartite structure of the chloroplast genome. A total of 135 genes were annotated, including 90 protein-coding genes, 37 transfer RNA (tRNA) genes, and eight ribosomal RNA (rRNA) genes, of which 20 genes were duplicated in the IR regions. Twelve cis-splicing genes and one trans-splicing gene, *rps12*, were identified (Supplemental Figures 2 and 3).

**Figure 3.**
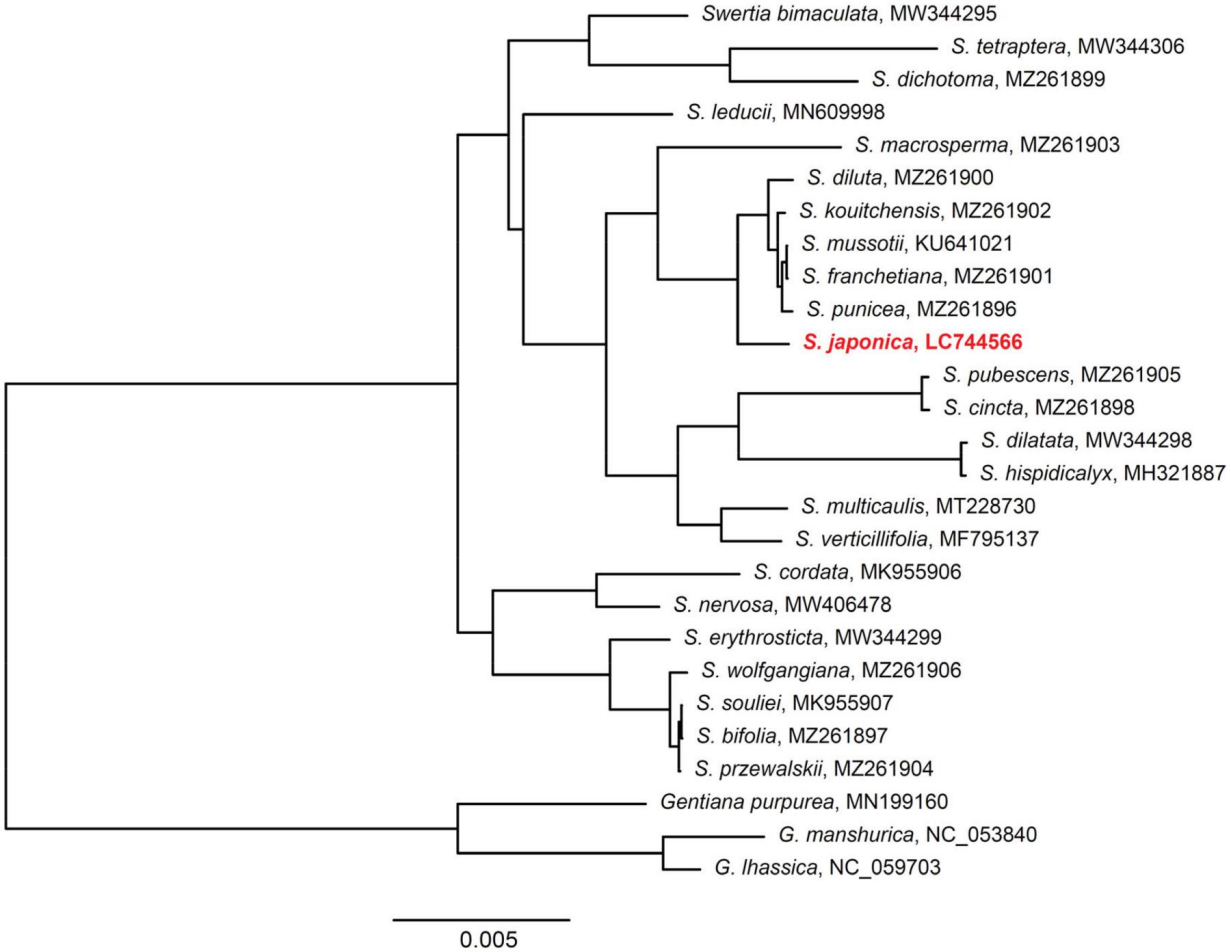
A maximum-likelihood (ML) tree of 24 *Swertia* species and three *Gentiana* species, which were used as outgroups. Bootstrap probabilities of all nodes are 100%. The genome of the species identified with red-bold font was presented in this study. The following sequences were used: *S. bimaculata* MW344295 (Xu et al. 2022), *S. tetraptera* MW344306 (Xu et al. 2022), *S. dichotoma* MZ261899 (Cao et al. 2022), *S. leducii* MN609998 (Yang et al. 2019), *S. macrosperma* MZ261903 (Cao et al. 2022), *S. diluta* MZ261900 (Cao et al. 2022), *S. kouitchensis* MZ261902 (Cao et al. 2022), *S. mussotii* KU641021 (Xiang et al. 2016), *S. franchetiana* MZ261901 (Cao et al. 2022), *S. punicea* MZ261896 (Cao et al. 2022), *S. pubescens* MZ261905 (Cao et al. 2022), *S. cincta* MZ261898 (Cao et al. 2022), *S. dilatata* MW344298 (Xu et al. 2022), *S. hispidicalyx* MH321887, *S. multicaulis* MT228730 (Zhang et al. 2020), *S. verticillifolia* MF795137, *S. cordata* MK955906 (Huang et al. 2019), *S. nervosa* MW406478, *S. erythrosticta* MW344299 (Xu et al. 2022), *S. wolfgangiana* MZ261906 (Cao et al. 2022), *S. souliei* MK955907, *S. bifolia* MZ261897 (Cao et al. 2022), *S. przewalskii* MZ261904 (Cao et al. 2022), *G. purpurea* MN199160, *G. manshurica* NC_053840 (Liang et al. 2020), and *G. lhassica* NC_059703 (Fu et al. 2021).

The phylogenetic analysis showed that 24 *Swertia* species were monophyletic with the highest probability (Figure 3). *Swertia japonica* was closest related to the clade consisting *S. diluta*, *S. franchetiana*, *S. kouitchensis*, *S. mussotii*, and *S. punicea* (Figure 3).

## Discussion and conclusions

The *S. japonica* chloroplast genome was similar in structure to the chloroplast genome of other *Swertia* species (Xiang et al. 2016; Huang et al. 2019; Cao et al. 2022; Xu et al. 2022; Yang et al. 2022). The result of the phylogenetic analysis in this study concur with the topology of *Swertia* (without the inclusion of *S. japonica*) presented in the previous study (Yang et al. 2022). The phylogenetic analysis suggests that *S. japonica* is closest related to the clade comprising *S. diluta*, *S. franchetiana*, *S. kouitchensis*, *S. mussotii*, and *S. punicea*. Among these relatives, *S. japonica*, *S. mussotii*, and *S. diluta* are prevalent in traditional Chinese and Japanese medicine (Xiang et al. 2016).

This study provides a reliable chloroplast genome of *S. japonica*. The presented complete chloroplast genome can contribute to taxonomic, pharmacological, and evolutionary studies that focus on *Swertia* and related genera.

## Supplementary Material

Supplemental Material

## Data Availability

The data that support the findings presented in this study are openly available in GenBank (NCBI, https://www.ncbi.nlm.nih.gov) under the reference number LC744566. The associated BioProject, SRA, and Bio-Sample numbers are PRJDB15262, DRR441012, and SAMD00578002, respectively.
