# Supplementary material for: The complete chloroplast genome sequence of Swertia japonica (Schult.) Makino (Gentianaceae)
Source: Mitochondrial DNA B Resour. 2023 Nov 1;8(11):1179–82. doi: 10.1080/23802359.2023.2275335 (PMC10769539; doi:10.1080/23802359.2023.2275335)
Supplement: Supplemental Material [file TMDN_A_2275335_SM0641.docx]

**Supplementary Figure 1** Read coverage depth map of the assembled genome.


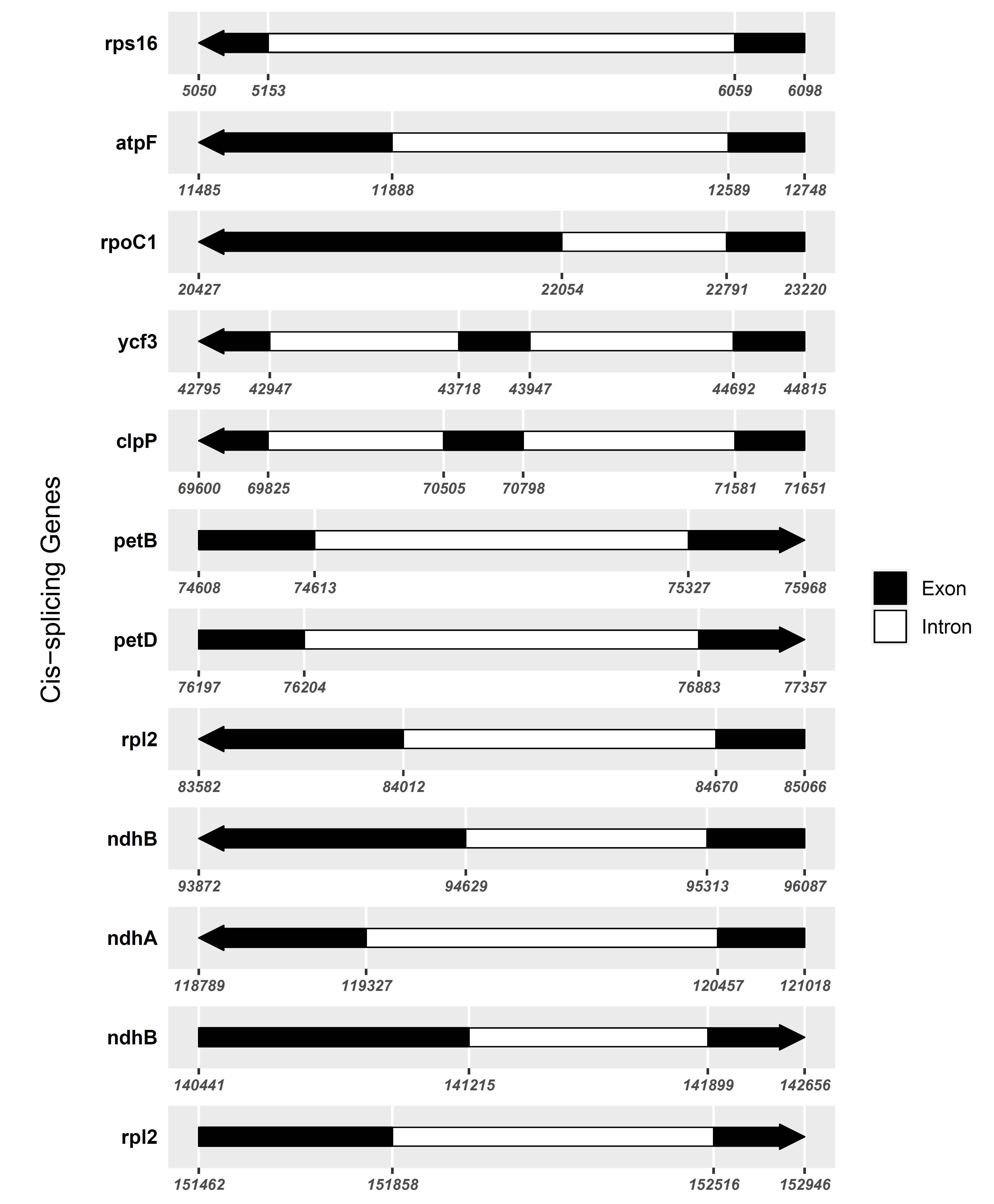


**Supplementary Figure 2** Schematic map of the cis-splicing genes in the chloroplast genome. Exons and introns are shown in black and white, respectively. The arrow indicates the sense direction of the gene. We noted that lengths of exons and introns are not drawn to scale.

**Supplementary Figure 3** Schematic map of the trans-splicing gene *rps12* in the chloroplast genome.
